# Modular organization of the white spruce (*Picea glauca*) transcriptome reveals functional organization and evolutionary signatures

**DOI:** 10.1111/nph.13343

**Published:** 2015-02-27

**Authors:** Elie S. M. Raherison, Isabelle Giguère, Sébastien Caron, Mebarek Lamara, John J. MacKay

**Affiliations:** ^1^ Center for Forest Research and Institute for Integrative and Systems BiologyUniversité LavalQuébecQCG1V 0A6Canada; ^2^ Department of Plant SciencesUniversity of OxfordOX1 3RBOxfordUK

**Keywords:** conifers, modular organization, tissue differentiation, transcriptional network, transcriptome, white spruce (*Picea glauca*)

## Abstract

- Transcript profiling has shown the molecular bases of several biological processes in plants but few studies have developed an understanding of overall transcriptome variation. We investigated transcriptome structure in white spruce (*Picea glauca*), aiming to delineate its modular organization and associated functional and evolutionary attributes.
- Microarray analyses were used to: identify and functionally characterize groups of co‐expressed genes; investigate expressional and functional diversity of vascular tissue preferential genes which were conserved among *Picea* species, and identify expression networks underlying wood formation.
- We classified 22 857 genes as variable (79%; 22 coexpression groups) or invariant (21%) by profiling across several vegetative tissues. Modular organization and complex transcriptome restructuring among vascular tissue preferential genes was revealed by their assignment to coexpression groups with partially overlapping profiles and partially distinct functions. Integrated analyses of tissue‐based and temporally variable profiles identified secondary xylem gene networks, showed their remodelling over a growing season and identified *PgNAC‐7* (no apical meristerm (NAM), *Arabidopsis* transcription activation factor (ATAF) and cup‐shaped cotyledon (CUC) transcription factor 007 in *Picea glauca*) as a major hub gene specific to earlywood formation.
- Reference profiling identified comprehensive, statistically robust coexpressed groups, revealing that modular organization underpins the evolutionary conservation of the transcriptome structure.

## Introduction

Transcriptome analysis is among the most widely used large‐scale experimental approaches to investigate genome function and to help annotate genome sequences (Pickrell *et al*., 2010; Prasad *et al*., 2013; Ellison *et al*., 2014). The transcriptome as a whole is highly dynamic as a consequence of quantitative variations over spatial and temporal scales. The observed changes in transcript levels result from the combinatorial action of genetically encoded regulatory pathways that orchestrate development and responses to environmental stimuli. The accumulation of individual gene transcripts may be viewed as a bridge between the genotype and the phenotype and, as such, may be acted upon through natural selection (Romero *et al*., 2012; Prasad *et al*., 2013).

The fundamental interest in and application of transcriptome analysis are reflected in its broad application in nonmodel organisms, including many taxa such as conifer trees where large genome size or complexity represents a technical barrier to genomic investigation. Conifer tree genomes are highly repetitive and, with sizes of 18–30 Gb, they are several fold larger than most angiosperm plant genomes sequenced to date, including *Arabidopsis thaliana* (Arabidopsis Genome Initiative, 2000), black cottonwood (*Populus trichocarpa*; Tuskan *et al*., 2006), maize (*Zea mays*; Schnable *et al*., 2009), and eucalyptus (*Eucalyptus grandis*; Myburg *et al*., 2014) as well as animal genomes such as human (IHGSC, 2004). Three conifer genomes were recently sequenced and include white spruce (*Picea glauca*; Birol *et al*., 2013), Norway spruce (*Picea abies*; Nystedt *et al*., 2013), and loblolly pine (*Pinus taeda*; Neale *et al*., 2014). By contrast, cDNA and transcriptome sequencing in conifers has been ongoing for over 15 yr (Allona *et al*., 1998), has led to many functional investigations, and produced outcomes that enable genetic mapping research, association studies and investigations of evolutionary adaptation (MacKay *et al*., 2012). Despite this extensive body of work, a general framework describing transcriptome organization in conifers is lacking and would help to develop a more unified understanding of genome function.

Conifer transcriptome studies have been conducted to gain insight into the molecular underpinnings of growth, development and stress responses. Conifers are long‐lived and need to cope with diverse biotic and abiotic stress factors over time, which has led to a number of studies on the theme of resistance and acclimation. For example, Sun *et al*. (2011) identified genes responsive to fungal elicitation in Scot's pine (*Pinus sylvestris*) while Verne *et al*. (2011) identified up‐ and down‐regulated genes in white pine weevil‐resistant individuals in interior spruce (*P. glauca* × *Picea engelmannii*). In the context of global environmental change, Yeaman *et al*. (2014) investigated patterns of gene expression related to acclimation across environmental conditions in lodgepole pine (*Pinus contorta*) and interior spruce; and Baldi *et al*. (2011) identified candidate genes for cold tolerance in cypress (*Cupressus sempervirens*). Considering the ecological and economic importance of conifer woods, another area of interest is transcriptome variation and reorganization during secondary xylem growth. Genes with significant changes in expression between earlywood and latewood formation were identified in loblolly pine trees from two seed sources (Yang & Loopstra, 2005), in maritime pine (*Pinus pinaster*) during a growing season (Paiva *et al*., 2008), and in radiata pine (*Pinus radiata*) at different stages of developmental aging (Li *et al*., 2010). A few studies have addressed related evolutionary aspects by identifying genes that are conserved in expression patterns and sequence between species such as white spruce and *A. thaliana* (Pavy *et al*., 2008), among different spruce species (Raherison *et al*., 2012) and between pine and spruce (Yeaman *et al*., 2014).

A majority of transcriptome profiling experiments in conifers and plants in general have aimed to identify and functionally describe expression variations in subsets of genes associated with biological processes or genotypic differentiation (Lorenz *et al*., 2011; Palle *et al*., 2011). Genes whose transcript levels do not vary across samples have been less investigated, despite their potentially key housekeeping roles (Aceituno *et al*., 2008; Chang *et al*., 2011; Eisenberg & Levanon, 2013). Furthermore, many studies have focused on a single or a few tissues or organs; however, recent studies in flowering plants show that tissue differentiation is the major driver of transcriptome reorganization in species such as *A. thaliana* (Ma *et al*., 2005), maize (Downs *et al*., 2013), barley (*Hordeum vulgare*; Downs *et al*., 2013), and tobacco (*Nicotiana tabacum*; Druka *et al*., 2006). The conservation of tissue‐specific gene expression patterns is also well established in vertebrates including mammals (Prasad *et al*., 2013) and nonmammals (Chan *et al*., 2009). Taken together, these observations indicate that studies considering several tissues are a powerful approach to delineate transcriptome organization across a whole organism. Furthermore, the rate of gene expression evolution can differ depending on tissue or organ sources in angiosperms (Yang & Wang, 2013) and in mammals (Brawand *et al*., 2011).

We investigated transcriptome structure by interrogating 23 853 genes on a custom‐designed microarray profiling platform (Raherison *et al*., 2012) in two different controlled experiments with biological replications. We have previously shown a high level of concordance between results obtained with this microarray and RNA‐Seq data (Raherison *et al*., 2012). Other recent studies used RNA‐Seq profiling (Yeaman *et al*., 2014) and were able to interrogate nearly identical numbers of genes (23 889 in spruce and 23 519 in pine). Our objectives were threefold. (1) We first aimed to characterize the organization of the white spruce transcriptome based on multiple tissue data generated from a single experiment enabling statistical model analyses and the identification of high‐confidence coexpression groups. This led us to define and functionally analyse expressional groups including invariant and variable genes across vegetative tissues of white spruce. (2) Our second objective was to investigate expressional and functional diversity of vascular tissue preferential genes which were conserved among *Picea* species. (3) Our third objective was to delineate gene coexpression networks and identify genes involved in secondary cell wall development during wood formation, and study network reorganization over the course of a growth season.

## Materials and Methods

We used microarray data sets from two independent experiments (Expts 1 and 2) using the same microarray platform as described in Raherison *et al*. (2012). These experiments are described in the Plant material section. We also used gene expression results in shoot secondary xylem and phelloderm tissues of white spruce (*Picea glauca* (Moench) Voss), black spruce (*Picea mariana* (M) Britton) and Sitka spruce (*Picea sitchensis* (Bong.) Carrière) previously described in Raherison *et al*. (2012). Unless specified otherwise, all statistical analyses were performed using the base R packages; the Benjamini & Hochberg method was employed where appropriate to correct sets of *P*‐values for multiple comparisons (Benjamini & Hochberg, 1995) using the multtest R package, and the hypergeometric test for under‐ or overrepresentation analysis.

### Plant material

**Figure 1:**
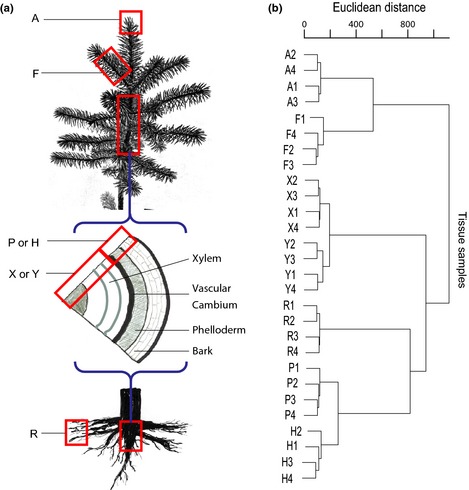
Classification of collected tissues based on gene expression. (a) Tissue sampling (image provided by Dr A. Séguin). (b) Hierarchical clustering of white spruce (*Picea glauca*) tissues based on microarray data (log_2_ scale). The analysis used the *hclust* function of R with Ward's method (Ward, 1963) and based on expression data of detected genes. A, shoot apex; F, young foliage; X, shoot secondary xylem; Y, root secondary xylem; R, root tips; P, shoot phelloderm; H, root phelloderm. Each tissue was replicated four times (1–4).

We used young spruce trees from open‐pollinated seed lots that were grown for 6–16 wk in a glasshouse under natural light conditions. Tissue samples were collected either at one (Expt 1) or at four different time‐points (Expt 2) in a growing season. In Expt 1, in July we collected from 3‐yr‐old seedlings of white spruce seven vegetative tissues: shoot apex, needles of young branches (young foliage), shoot or root secondary xylems, shoot or root phelloderms, and root tips (Fig. 1a). The shoot apex sample was *c*. 1 cm in length and contained the shoot tip with the apical bud as well as primary needles. Secondary xylem and phelloderm tissues lay inside and outside the vascular cambium, respectively (Raherison *et al*., 2012). Four biological replicates were used for each tissue sample, and each replicate was a pool of samples from five independent trees. In Expt 2, shoot secondary xylem tissues of 4‐yr‐old seedlings of Norway and white spruces were sampled on 22 June, 13 July, 17 August and 21 September. Samples were collected from six individual trees for each time‐point. All of the samples were immediately frozen in liquid nitrogen and stored at −80°C until RNA isolation.

### RNA isolation, labelling and hybridization

Total RNA was isolated from tissue samples using the cetyltrimethyl ammonium bromide (CTAB) extraction method of Chang *et al*. (1993) as described in Pavy *et al*. (2008). Total RNA of each sample replicate was amplified using the Amino Allyl MessageAmp II aRNA amplification kit (Ambiom by Life Technologies, Carlsbad, CA, USA) following the manufacturer's instructions. In Expt 1, dye‐swap technical replicates for each biological replicate were performed. Each sample of amplified RNA (aRNA) was paired and labelled with Alexa Fluor 555 and 647 dyes (Invitrogen, Carlsbad, CA, USA). In Expt 2, aRNAs were labelled with Alexa Fluor 555 dye. RNA amplification, aRNA labelling, hybridization, array scanning and image processing were performed as described in Raherison *et al*. (2012).

### Microarray preprocessing and statistical analyses

In Expt 1, intensity data for single dyes of the two‐dye experiment were analysed separately. Replacement of abnormal or flagged spots, data normalization, and identification of positive probes and detected genes were performed as described in Raherison *et al*. (2012). A total of 24 850 probes corresponding to 23 623 unique white spruce genes were detected at least in one tissue (Table 1, Supporting Information Table S1). Using the nlme R package, we applied to detected genes a mixed analysis of variance (ANOVA) model with tissue, dye and the interaction tissue × dye as fixed effects, and the interaction tree × tissue as a nested random effect. A set of 846 genes were not included in the ANOVA because their expression levels were nonnormally distributed (Anderson–Darling's test; nortest R package), had nonhomogenous variances (Levene's test; car R package), or were affected by the factor dye or the interaction tissue × dye. We identified genes that had changes (variable genes; adjusted *P*‐value < 0.05) or were unchanged (invariant genes; adjusted *P*‐value > 0.05) across expression tissues from the remaining detected genes. These remaining detected genes were also used for further analyses. In Expt 2, flagged spots of expression data were removed from the analysis. Data normalization and identification of positive probes were carried out as described in Raherison *et al*. (2012). A two‐way ANOVA with species and time as factors was performed to identify the 1366 temporally variable genes or genes differentially expressed in secondary xylem across time‐points in a growing season. Temporally variable genes were those with one or more statistically significant probes (adjusted *P*‐value < 0.05). The average expression values in two dye technical replicates for each tissue sample (Expt 1) or six replicates in two species for each time‐point (Expt 2) were used for weighted correlation network analysis (wgcna R package; Langfelder & Horvath, 2008), template matching (Harris *et al*., 2009), and correlational and hierarchical clustering analyses. In wgcna, we used soft‐thresholding power values of 14 and 18 for Expts 1 and 2, respectively, to identify expression modules. An expression module is a cluster of highly (positively and negatively) correlated genes. It reveals two negatively correlated expression profiles. Each expression profile is associated with a group of genes that we referred to as a coexpression group in Expt 1 and a temporally variable cluster in Expt 2 (Table S1). The wgcna script used is included in Methods S1. The raw and preprocessed data have been deposited in the National Center for Biotechnology Information – Gene Expression Omnibus (NCBI‐GEO) with accession numbers GSE60277 and GSE51884 for Expts 1 and 2, respectively.

**Table 1:** Numbers of genes detected and classification as variable and invariant based on transcript levels

Category	Total genes	Annotated genes (%)^a^
Interrogated genes	23 853	15 724 (66)
Detected genes^b^	23 623	15 586 (66)
Statistically tested genes (ANOVA)^c^	22 857	15 096 (66)
Invariant genes	4805	2410 (50)
Variable genes	18 052	12 726 (70)
High‐confidence genes (adjusted *P*‐value < 0.0001)	10 548	7651 (73)
Low‐confidence genes (0.05 < adjusted P‐value > 0.0001)	7504	5075 (68)

a Genes that matched an *Arabidopsis thaliana* sequence (BLASTX; *E*‐value < 10^−10^).

b Transcripts were detected above background levels in at least one tissue of white spruce (*Picea glauca*).

c Genes that met assumptions for ANOVA testing (for details, see the Materials and Methods section).

### Functional annotation analysis

The cDNA sequences from which the oligonucleotide probes were designed (Raherison *et al*., 2012) were annotated against the *A. thaliana* protein data set (The Arabidopsis Information Resource 10) using BLASTX (*E*‐value < 10^−10^). Functional classes presented in Table S2 were developed as follows: first, we identified and clustered enriched biological process gene ontology (GO) terms using the Database for Annotation, Visualization and Integrated Discovery (DAVID; enrichment at *P*‐value < 0.05; Huang *et al*., 2009); second, enriched GO terms were classified into functional categories of plant GO slim using categorizer (Hu *et al*., 2008); and finally, based on the GO term itself, its cluster and its functional category, each GO term was manually assigned to a consensus functional class. Six sequences that we analysed represented *P. glauca* MYB proteins. These sequences were named in this report: BT112255, PgMYB29; BT117714, PgMYB30; BT119291, PgMYB31; DR571012, PgMYB32; BT108182, PgMYB33; and BT106711, PgMYB34.

### Reverse transcription quantitative PCR (RT‐qPCR)

We performed RT‐qPCR on 28 genes to verify results from microarray analyses (Table S3). Gene‐specific primers were designed using primer3plus (Untergasser *et al*., 2007), evaluated with oligocalc (Kibbe, 2007) and checked for uniqueness in the white spruce gene catalogue (Rigault *et al*., 2011). The same RNA samples that were used for microarray experiments were used for reverse transcription and RT‐qPCR as described in Boyle *et al*. (2009) using the QuantiFast SYBR Green PCR Kit (Qiagen, Carlsbad, CA, USA) with 5 ng of cDNA in a final volume of 15 μl. The number of molecules was determined following the method of Rutledge & Stewart (2008) with modifications described in Boyle *et al*. (2009). A one‐way ANOVA (for the factor tissue) and the template matching method were used to determine whether results from microarray and RT‐qPCR analyses were statistically matched with regard to gene category and expression profile, respectively.

## Results

### Most white spruce genes vary significantly across vegetative tissues

We analysed the transcript levels of 23 853 unique genes in seven vegetative tissues during normal development of white spruce (Expt 1). These tissues represent aboveground (apex, young foliage or needles, shoot secondary xylem, and shoot phelloderm) and belowground (root secondary xylem, root phelloderm and root tips) organs. Microarray analysis of each tissue sample comprised four biological replicates that were analysed separately. Dye‐swap technical replicates were also used. We identified 18 052 genes whose expression level varied significantly across tissues (variable genes) and 4805 whose expression level did not vary across tissues (invariant genes) (Tables 1, S1, S4). A total of 10 548 variable genes were identified as high confidence (adjusted *P*‐value < 0.0001) and 7504 genes (0.0001 < adjusted *P*‐value < 0.05) as low confidence (Tables 1, S1, S4).

### The white spruce transcriptome is organized into coexpression groups classifying tissues based on their types or physiological functions

As a first step to explore expression data and transcriptome organization, we used hierarchical clustering. Biological samples of each tissue were clustered within a single unique group, thus indicating high repeatability of transcript level determinations as well as uniformity among samples of each type (Fig. 1b). Identical results were obtained when analysing either the whole set of detected genes (Fig. 1b) or only the set of variable genes (Fig. S1). The clustering clearly formed groups according to tissue type. Root xylem clustered closely with shoot xylem, and root phelloderm with shoot phelloderm, thus indicating a strong similarity between tissues from above‐ and belowground organs. The cluster mostly similar to foliage (i.e. needles) was the shoot apex, an organ that contains primordial leaves.

**Figure 2:**
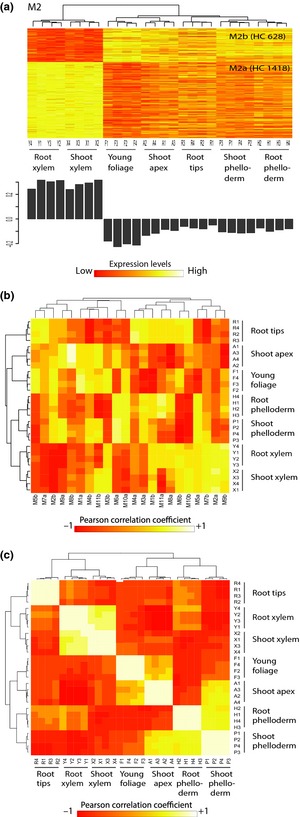
Expression module and coexpression groups across tissues in white spruce (*Picea glauca*). (a) Heatmaps show transcript abundance (log_2_ scale) of high‐confidence variable genes in the M2 expression module and M2a,b coexpression groups determined with the wgcna package of R. Number in parentheses, number of high‐confidence (HC) variable genes in the coexpression group. Total and low‐confidence variable gene numbers are listed in Supporting Information Table S4. Rows (*y*‐axis), genes which are listed in Table S1; columns (*x*‐axis), each of the four replicates of tissues (P1–4, shoot secondary phelloderm; Y1–4, root secondary xylem; R1–4, root tip; X1–4, shoot secondary xylem; F1–4, young foliage; A1–4, shoot apex; H1–4, shoot secondary phelloderm). Bar plots display the eigengene modules (or the first principal components). See Fig. S2 for a graphical representation of other expression modules and coexpression groups. (b) Correlation between coexpression groups (M1a–M11b) and tissues based on eigengene groups. (c) Correlation between tissues based on eigengene groups.

To further characterize the organization of the white spruce transcriptome, we clustered variable genes and identified 22 coexpression groups or sets of genes sharing a similar expression profile, ranging in size from 48 to 1929 genes (Figs 2a, S2; Tables S1, S4). Gene clustering to delineate the coexpression groups proceeded in two steps. First, we clustered high‐confidence genes into 11 modules each containing two coexpression groups with the wgcna package of R and defined the expression profile associated with each of the groups. Second, we used the resulting average expression level within each of the 22 groups as a template to determine membership of low‐confidence genes within the coexpression groups (adjusted *P*‐value < 0.05). In this study, expression modules (M1–M11) are comprised of genes whose expression was positively or negatively correlated, whereas members of a coexpression group had only positively correlated profiles (M1a–M11b; 22 groups in total), as shown in heatmaps (Figs 2a, S2a–j). A principal component analysis (eigengene modules) was used to show the differentiation of tissues within each expression module (Figs 2, S2). Eigengene groups were used to study correlation between groups and tissues (Figs 2b, S2k) and between tissues (Fig. 2c).

Our results indicate a modular organization of the transcriptome that is linked to differentiation between tissue types or physiological functions (Figs 2a,b, S2; Table S4). For example, four coexpression groups containing 42% of the variable genes were positively correlated with a single tissue (average Pearson correlation coefficient *R *>* *0.7; shoot apex in M4a, M8b, M9a and M11b; and needles in M5b) or tissue type (*R *>* *0.6; secondary xylem from shoots and roots in M2a, M7b and M9b; and phelloderm from shoots and roots in M6a and M10a). The profile of the M1a group comprising 11% of the variable genes was positively correlated with photosynthetic tissues (*R *=* *0.8).

The identification of coexpression groups also supports a strong similarity between tissues from above‐ and belowground organs. For example, 14 profiles comprising 70% of variable genes closely clustered xylem samples from the shoot and the root, and similar phelloderm samples from the shoot and the root (M2–M7 and M10). By contrast, only 21% of variable genes were separately clustered for vascular tissues from the root and the shoot (M1 and M11). Analysis based on eigengene groups revealed highly positive correlation between vascular tissues from the shoot and the root (Fig. 2c).

To confirm microarray analysis results, we determined and statistically analysed transcript levels of selected genes using RT‐qPCR, including 24 variable genes and four invariant genes (Table S3). Results were strongly consistent between the two methods, with 92% of tested genes having similar expression profiles and similar categories (invariant and variable genes) (Table S3).

### GO term enrichments show functional diversity among coexpression groups

**Figure 3:**
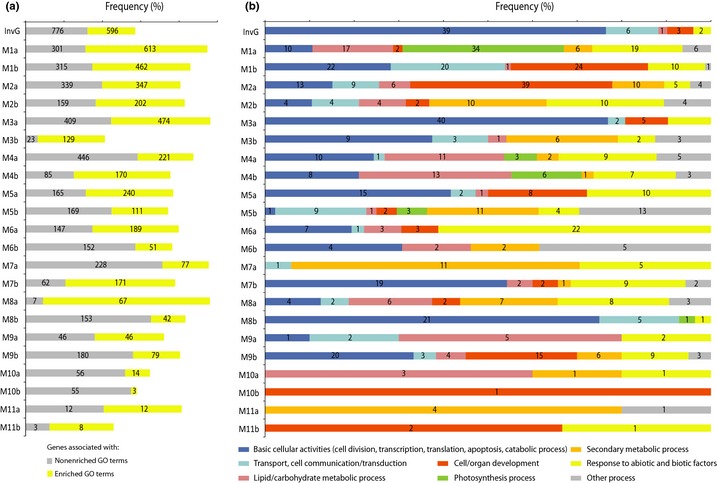
Functional analysis of invariant genes (InvG) and coexpression groups (M1a–M11b) in white spruce (*Picea glauca*). (a) *x*‐axis, frequency (%) of genes associated with each of the two annotation categories: enriched and nonenriched biological process gene ontology (GO) terms. Numbers inside bars are the number of genes per category. (b) *x*‐axis, frequency (%) of enriched GO terms associated with consensus functional classes. Numbers inside bars are the number of enriched GO terms per consensus functional class.

Functional analysis was performed for the 22 coexpression groups and invariant genes based on annotations and enrichment for biological process GO terms for genes. Most of the detected genes (66%) were similar to an *A. thaliana* gene sequence (Table 1) and were annotated accordingly. The proportion of white spruce sequences that matched an *A. thaliana* sequence was somewhat higher among variable genes (70% for the high‐confidence group) than for the invariant genes (50%; Table 1). We found 4324 genes associated with enriched GO terms, representing 50% of the genes with assigned GO term annotations (Figs 3a, S3; Table S2).

The GO term enrichment results showed that the dominant enriched biological processes varied across the coexpression groups (Fig. 3b). In addition, strong or preferential expression in a tissue type may be associated with several coexpression groups from different modules, and thus with enrichment of different processes. For example, group M6a with genes strongly expressed in phelloderm tissues compared with all other tissues was enriched for stress response. Group M10a was also highly expressed in phelloderm tissues but contrasted with M6a through stronger expression in needles; it was enriched for terms related to cell/organ development. Observations such as these show how assignment to a coexpression group is a useful first step for developing hypotheses for different groups of phelloderm preferential genes. Other similar examples were observed for groups of genes with strong expression in apex tissue (M4a, M8b, M9a and M11b) or in xylem tissues (M2a, M7a and M9b). These findings revealed the diversity of functions among genes preferentially expressed in a tissue type.

### Conifer‐specific genes are more strongly associated with secondary meristematic tissues

Functional and structural differences have been extensively documented in secondary vascular tissues when comparing conifer and angiosperms trees. For example, functions of water transport and mechanical support are attributed to a single cell type in conifers (tracheids) and to two distinct cell types in angiosperms (vessels and fibres); in addition, different types of hemicelluloses and lignins make up their secondary cell walls. We thus explored to what extent genes that are identified as unique or specific to conifers may be more represented in coexpression groups with strong expression in these tissue types. We used results of searches for sequence similarity to *A. thaliana* sequences (this work) and to Sitka spruce and loblolly pine sequences (Rigault *et al*., 2011) to identify putative conifer‐specific genes and analysed their distribution across coexpression groups. We found that conifer‐specific genes were overrepresented in groups with a preference for expression in secondary vascular tissues (M2a and M6a), and underrepresented in groups without a clear preference in any tissue (M1b and M3a) or with high expression in tissues more closely derived from the shoot meristem (M1a and M8b) (Table S5).

### Highly conserved invariant genes are associated with high expression and basic cell functions

We observed that invariant genes spanned a broad range of expression levels (Table 2) and that fewer of them had functional annotations compared with the variable genes (Figs 3a, S3). Overall, the 596 invariant genes within enriched GO terms were predominantly associated with basic cellular activities and to a lesser extent with transport, cell communication/transduction, cell/organ development, and stress response (Fig. 3b), suggesting an overrepresentation of housekeeping genes. We analysed the group further with the aim of uncovering relationships between expression levels and functional annotations or sequence similarities. Results showed that genes that were conserved between conifers and *A. thaliana* were more strongly expressed as they were overrepresented among the high‐expression classes. Conversely, the putative conifer‐specific genes were overrepresented in low‐expression classes (Table 2). Genes associated with the major enriched functions were distributed in similar proportions in all expression level classes except for the stress response category, which was statistically overrepresented in the lowest expression class. This latter group probably represents genes whose expression may vary in response to a stress factor and had a low basal level in tissues grown in permissive conditions.

**Table 2:** Function and cross‐species conservation of invariant genes according to their expression levels

Expression classes^a^	Total	Cross‐species sequence conservation^b^			Functional classes^c^			
		Conifers	Conifers–*Arabidopsis thaliana*	White spruce–*Arabidopsis thaliana*	A (%)	B (%)	C (%)	D (%)
Low (< Q1)	1202	**142** *	*225* *	*110* *	79	7	**16** *	11
Low to moderate (Q1, Q2)	1201	**147** *	*227* *	*89* *	94	6	8	10
Moderate to high (Q2, Q3)	1201	109	**419** *	*134* *	94	10	7	13
High (> Q3)	1201	*82* *	**686** *	**397** *	90	9	10	20
Total in number	4805	480	1557	730				

a Based on their mean expression from all tissue samples of white spruce (*Picea glauca*), invariant genes were divided into four equal classes delimited by the first, second and third quartiles (Q1, Q2 and Q3, respectively).

b Conifers: number of genes with conserved sequences between white spruce (*Picea glauca*), sitka spruce (*P. sitchensis*) and loblolly pine (*Pinus taeda*); Conifers–*Arabidopsis thaliana*: number of genes with conserved sequences between one of the three conifer species and *A. thaliana*; and White spruce–*A. thaliana*: number of genes with conserved sequences between white spruce and *A. thaliana* based on the sequence similarity approach (Rigault *et al*., 2011; this work; BLASTX; *E*‐value < 10^−10^).

c Frequency (%) of genes associated with each of the functional classes: A, basic cellular activities; B, cell/organ development; C, response to abiotic and biotic factors; D, transport, cell communication/transduction. The sum of percentages per row exceeds 100% because a gene may be involved in one or more functional classes.

^a,b^Hypergeometric tests performed for expressional classes by column: *, statistically significant (adjusted *P*‐value < 0.05), for overrepresentation (bold numbers) or underrepresentation (italic numbers).

### Expressional and functional diversity among conserved secondary xylem and phelloderm preferential genes

**Figure 4:**
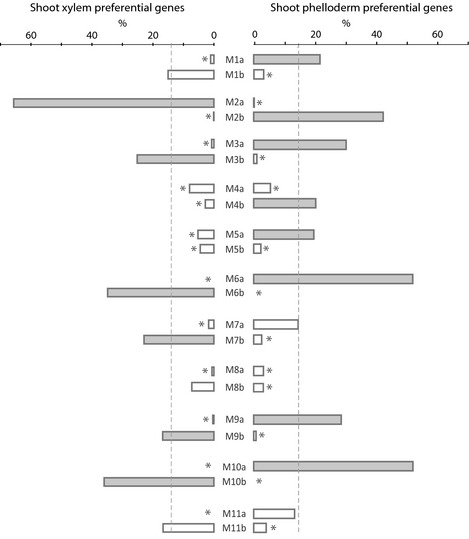
Distribution of secondary xylem or phelloderm preferential genes conserved among *Picea* species across coexpression groups. Vascular tissue preferential genes in white (*P. glauca*), Norway (*P. abies*) and black (*P. mariana*) spruces were identified in Raherison *et al*. (2012). Dashed grey vertical lines represent the expected frequency based on chance alone (%) of preferential genes across coexpression groups. Overrepresentation of xylem or phelloderm preferential genes compared with chance alone (hypergeometric test; adjusted *P*‐value < 0.05) is shown by closed bars and underrepresentation of xylem or phelloderm preferential genes is shown by open bars with an asterisk (hypergeometric test; adjusted *P*‐value < 0.05). Each of the over‐ and underrepresentations of xylem and phelloderm preferential genes closely matched with the expression profiles of the corresponding coexpression groups as shown in Fig. 2(a) and Supporting Information Fig. S2(a–j).

We used the defined coexpression groups to characterize sets of genes that we previously identified as preferentially expressed in secondary xylem (2537 genes) and phelloderm (2800 genes) in shoots and were conserved in white, black and Sitka spruces (Raherison *et al*., 2012; Table S1). We found that 95% of the conserved xylem and phelloderm preferential genes were distributed among all coexpression groups, indicating that their modular organization is represented by diverse expression profiles (Fig. 4). Their respective distributions among coexpression groups were highly contrasted and matched closely with the profiles of the coexpression groups with regard to expression in xylem and phelloderm tissues, except for group M1a (with similar expression levels in both xylem and phelloderm tissues). The two tissue types are derived from different but neighbouring secondary stem meristems; the xylem is derived from the cambial zone (located more internally) and the phelloderm is derived from the phellogen (located more externally). Our results show the extent of transcriptome reorganization resulting from differentiation pathways from these two meristems.

### Distinct expression patterns characterize wood formation at different stages during a growth season

**Figure 5:**
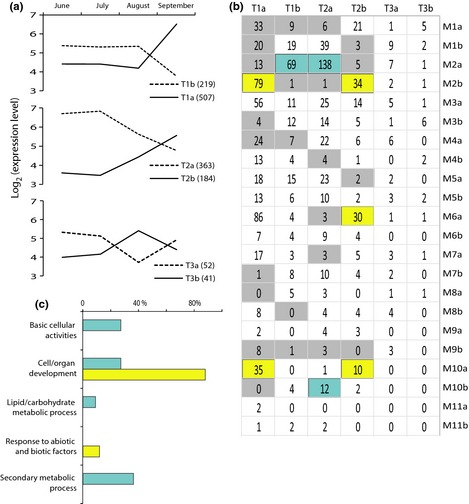
Distribution of temporally variable xylem expressed genes across coexpression groups. (a) Expression profiles of temporally variable genes in secondary xylem of white (*Picea glauca*) and Norway (*P. abies*) spruces. Numbers in brackets are the number of genes within temporally variable clusters (T1a–T3b). (b) Number of temporally variable genes across tissue‐based coexpression groups (M1a–M11b). Grey and blue/yellow areas, under‐ and overrepresentation (hypergeometric test; adjusted *P*‐value < 0.05), respectively; blue and yellow areas, earlywood and latewood genes, respectively, that is, June–July (T1b and T2a) and September (T1a and T2b) preferential genes that were overrepresented in M2a and M10b, and in M2b, M6a and M10a, respectively. (c) Functional annotation analysis based on gene ontology (GO) term enrichment of earlywood (blue bars) and latewood (yellow bars) genes.

Secondary vascular development and growth in woody plants follow an annual cycle that is characterized by an initial rapid growth phase (earlywood formation), followed by a slower phase that transitions to dormancy (latewood formation). We carried out a time‐course microarray profiling experiment (Expt 2) and identified 1366 genes with significant temporal variation in white and Norway spruces (adjusted *P*‐value < 0.05). The genes were clustered with wgcna into six temporal coexpression groups of 41–507 genes (Fig. 5a; Table S1). Overall, transcript levels did not change between June and July, remained stable (Fig. 5a; cluster T1; 726 genes) or varied progressively (Fig. 5a; cluster T2; 547 genes) in August and changed dramatically from August to September. Transcripts accumulating preferentially during June–July and September represented 51 and 43%, respectively, of the genes (Fig. 5a). By contrast, only 3% of the genes were preferentially expressed in August.

Next, we analysed the temporally variable gene clusters for membership of the 22 coexpression groups derived from our comparison of vegetative tissues in white spruce (Fig. 5b). We found that June–July preferential genes (average Pearson correlation coefficient *R *=* *0.6) were overrepresented in groups exhibiting high expression in xylem tissues (M2a and M10b); these genes are called earlywood genes. This observation is consistent with the fact that the secondary xylem tissues used to define the tissue‐based coexpression groups were sampled in July during earlywood formation (Fig. 5b). Conversely, September preferential genes (*R *=* *0.8) were overrepresented in groups with a strong preference in phelloderm tissues or in other tissues than xylem (M2b, M6a, and M10a); these genes are referred to as latewood genes (Fig. 5b). These observations indicate that many of the earlywood genes were specific to xylem tissues at the start of the growing season, while latewood genes were more active in other parts of the trees during early phases of the growth season. Analysis of functional annotations indicated that basic cellular activities, lipid/carbohydrate reserves and secondary metabolic process were specific to earlywood genes and that stress responses were specific to latewood genes. Cell/organ development is a common function with a higher representation in latewood genes (Fig. 5c; Table S2).

### Variation in secondary cell wall regulatory network hub genes is associated with xylem transcriptome reorganization during the growth season

We developed three unweighted and signed networks targeting two xylem preferential modules (M2 and M7), June–July and September preferential genes. In these analyses, two genes whose expression profiles had an absolute Pearson correlation coefficient above 0.9 were connected. We computed the number of genes connected to each gene (i.e. degree) to identify a hub(s), which represent(s) the most connected gene(s).

**Figure 6:**
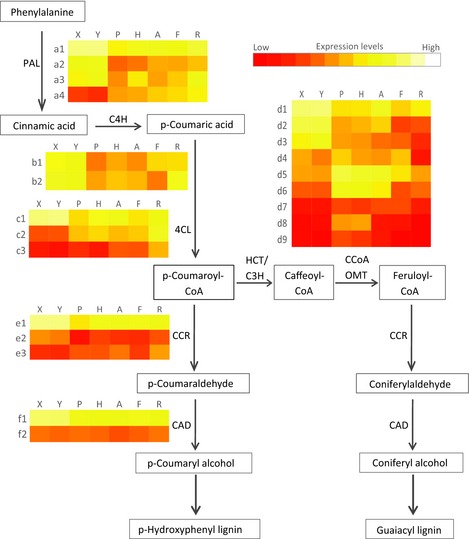
Phenylpropanoid pathway leading to lignin production. Boxes, metabolite; arrows, enzyme reactions; enzymes are reported using a three‐letter code: PAL, phenylalanine ammonia lyase; C4H, cinnamate 4‐hydroxylase; 4CL, 4‐coumarate‐CoA ligase; HCT/C3H, shikimate/quinate hydroxycinnamoyltransferase/p‐coumarate 3‐hydroxylase; CCoAOMT, caffeoyl‐CoA O‐methyltransferase; CCR, cinnamoyl‐CoA reductase; CAD, cinnamoyl alcohol dehydrogenease. Heatmaps next to enzyme name indicate expression value of genes (a1‐4, b1‐2, c1‐3, d1‐9, e1‐3, f1‐2) across tissues (X, Y, P, H, A, F, R); Each expression value is the average of four the biological replicates per tissue; a1‐4, BT112211 (GenBank ID), BT106538, BT119163, BT114680; b1‐2, BT116118, BT117086; c1‐3, BT106671, BT116171, DR551141; d1‐9, BT106698, BT106398, BT101243, BT117977, BT110631, BT102905, BT112333, BT104804, BT114515; e1‐3, DR573886, BT111802, BT112289; f1‐2, BT112280, BT116920; X, shoot secondary xylem; Y, root secondary xylem; P, shoot phelloderm; H, root phelloderm; A, shoot apex; F, young foliage; R, root tips.

We identified the enriched pathways for the M2–7 network, which are shown in Table S6. Among the enriched pathways, we investigated the branch of the phenylpropanoid biosynthesis pathway leading to lignin biosynthesis (Fig. 6). Based on gene expression and sequence similarity, 12 of the 23 represented genes were preferentially expressed in xylem tissues and candidate genes for lignin biosynthesis. The remaining genes may be involved in the biosynthesis of phenolic compounds for purposes other than secondary cell wall lignification or in other branches of the phenylpropanoid metabolic pathway.

**Figure 7:**
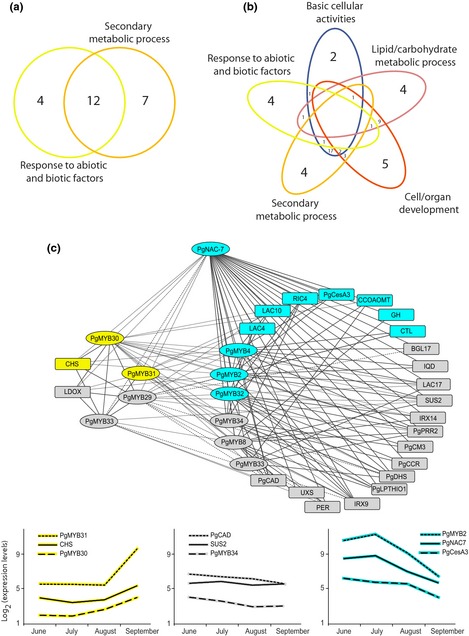
Functional annotations, network analysis and temporal expression patterns of *PgNAC‐7* (no apical meristerm (NAM), *Arabidopsis* transcription activation factor (ATAF) and cup‐shaped cotyledon (CUC) transcription factor 007 in *Picea glauca*) connected genes. (a, b) Venn diagrams show the distribution of *PgNAC‐7* (a) negatively and (b) positively connected genes among functional classes. (c) Upper panel, an unweighted and signed network shows connections of *PgNAC‐7* with MYB transcription factors (oval‐shaped nodes) and putative genes (rectangle‐shaped nodes) associated with secondary cell wall formation (Mizrachi *et al*., 2012; Duval *et al*., 2014). *PgNAC‐7*/MYB transcription factors and genes are listed in Supporting Information Table S1. LAC4, 10, 17, laccase4, 10 and 17; RIC4, rop‐interactive cdc42/rac‐interactive binding (CRIB) motif‐containing protein 4; CCOAMT, putative caffeoyl‐CoA O‐methyltransferase; GH, glycosyl hydrolase; CTL, chitinase‐like protein; BGL17, β‐glucosidase 17; IQD, protein IQ‐domain; SUS2, sucrose synthase 2; IRX9, 14, irregular xylem (probable β‐1,4‐xylosyltransferase) 9 and 4; PER, peroxidase; UXS, UDP‐xylose synthase; CHS, chalcone synthase; LDOX, leucoanthocyanidin dioxygenase; PgMYB2, 8, 29, 31, 32, 33, 34, MYB transcription factor 2, 8, 29, 31, 32, 33 and 34 in *P. glauca*; PgPRR2, pinoresinol reductase 2 in *P. glauca*; PgCesA3, cellulose synthase A 3 in *P. glauca*; PgCM3, chorismate mutase 3 in P. *glauca*; PgCCR, cinnamoyl CoA reductase in *P. glauca*; PgDHS, 3‐deoxy‐d‐arabino‐heptulosonate 7‐phosphate synthase in *P. glauca*; PgLPTHIO1, esterase/lipase/thioesterase family protein (lysophospholipase 1); PgCAD, cinnamyl alcohol dehydrogenase in *P. glauca*. Edges or connections are coexpression between genes with a Pearson correlation coefficient < −0.9 (dashed line) or ≥ +0.9 (solid line). Lower panel, temporal variation of genes; node and graph colours, temporal expression of genes in secondary xylem tissue of white (*Picea glauca*) and Norway (*P. abies*) spruces during a growing season: blue and yellow, highly expressed genes in June, July or August (earlywood genes) and in September (latewood genes), respectively; grey, temporally invariable genes.

The xylem preferential (M2–7) network was scale free (Fig. S4) and had *PgNAC‐7* (no apical meristerm (NAM), *Arabidopsis* transcription activation factor (ATAF) and cup‐shaped cotyledon (CUC) transcription factor 007 in *Picea glauca*) as the most connected hub gene (Table 3a). A subnetwork containing common interactions (connections) and genes between xylem (M2–7) and June–July preferential networks also had *PgNAC‐7* as the most connected hub gene (Tables 3a, S1). PgNAC‐7 was recently identified as a key regulator of secondary cell wall development in white spruce, which is functionally analogous to VND6/7 (vascular‐related NAC‐domain 6/7; Duval *et al*., 2014). Functional annotation analysis was carried out among genes positively and negatively connected to *PgNAC‐7*. Genes positively connected to *PgNAC‐7* were specifically enriched for basic cellular, development and reserve metabolisms, whereas stress response and secondary metabolism terms were enriched in both negatively and positively connected genes (Fig. 7a,b).

**Table 3:** Functional annotation of the top ten most connected genes (hubs) of the networks; (a) subnetwork (xylem (M2–7) and June–July preferential network) which contains 185 genes (see Supporting Information Table S1 for details); (b) September preferential network which contains 691 genes (see Table S1 for details)

GenBank accession no.	Cluster ID^a^	Degree^b^	Rank^c^	PFAM description^d^	Functional annotation^e^
(a)					
BT102049	GQ0165_B14	53	1	No apical meristem (NAM) protein	PgNAC‐7, NAC 007
CO235762	WS0021_I12	43	2		
BT117400	GQ03818_L16	40	3	Subtilase family	Subtilase family protein
DR591085	WS00830_F12	40	3		
BT108414	GQ03121_H22	39	5	Myb domain protein	PgMYB2*
BT106274	GQ02906_F17	36	6		
BT107150	GQ03103_E24	35	7	Xyloglucan endo‐transglycosylase (XET) C‐terminus; glycosyl hydrolase family	Xyloglucan endotransglucosylase/hydrolase
BT107836	GQ03113_F02	34	8	Aldehyde dehydrogenase family	Aldehyde dehydrogenase 3F1
DR594044	WS00839_J22	33	9	Subtilase family; protease‐associated domain; peptidase inhibitor I9	Subtilisin‐like serine endopeptidase family protein
BT106749	GQ03009_M04	32	10		Ring/U‐box superfamily protein
(b)					
BT105806	GQ02828_L20	496	1	Tubby C 2 (TUB)	Protein of unknown function (DUF567)
BT112527	GQ03316_N22	496	1	Leucine‐rich repeat	ADR1, disease resistance protein (CC‐NBS‐LRR class) family
BT117952	GQ03918_F10	496	1	Dehydrin (DHR)	
BT103744	GQ02801_H14	495	4	ThiF family	Ubiquitin‐activating enzyme
BT109195	GQ03205_L14	494	5	Glycosyl hydrolase family, N‐terminal domain	Glucuronidase
BT113137	GQ03326_D07	494	5	Dehydrin	
BT114680	GQ03519_E06	494	5	Aromatic amino acid lyase	Phenylalanine ammonia‐lyase 4
BT104565	GQ02811_O09	493	8	Embryo‐specific protein 3 (ATS3)	Embryo‐specific protein 3 (ATS3)
BT115870	GQ03707_L13	493	8	Glycosyl hydrolase family	β‐glucosidase
BT101047	GQ0065_L03	492	10		
BT104876	GQ02816_C10	492	10	Hsp20/alpha crystallin family	Heat shock protein 17.4
BT104881	GQ02816_D12	492	10		Sucrose synthase 3
EX329848	GQ02830_C21	492	10	Dehydrin (DHR)	
BT114833	GQ03603_F10	492	10	Dehydrin (DHR)	
BT116286	GQ03715_F15	492	10	Pyruvate phosphate dikinase, PEP/pyruvate binding domain	Pyruvate phosphate dikinase, PEP/pyruvate binding domain
DR574449	WS00737_M23	492	10		Cox19 family protein (CHCH motif)

a Rigault *et al*. (2011).

b The number of genes connected to each gene within the network.

c The rank of genes based on their degree within the network; genes with similar degrees have the same rank.

d PFAM, the protein families database; ATS3, *Arabidopsis thaliana* seed gene 3; PEP, phosphoenolpyruvate.

e Functional annotation based on The Arabidopsis Information Resource version 10 (TAIR10); DUF, domain of unknown function; NAC 007, no apical meristerm (NAM), Arabidopsis transcription activation factor (ATAF) and cup‐shaped cotyledon (CUC) transcription factor 007; PgNAC‐7, name of the NAC 007 gene in white spruce (*Picea glauca*) according to Duval *et al*. (2014); ADR1, activated diesease resistance 1; CC‐NBS‐LRR, coiled coil–nucleotide‐binding‐site–leucine‐rich‐repeat.

We present a network showing *PgNAC‐7* and genes involved in secondary cell wall formation as reviewed in Mizrachi *et al*. (2012) or shown to be transactivated in conifers by regulators of secondary cell wall formation including MYB transcription factors (Bomal *et al*., 2008; Duval *et al*., 2014; Fig. 7c; Table S7). The network includes temporally variable and nonvariable genes (Fig. 7c). Temporally variable genes included *PgNAC‐7*, five of the 10 MYB genes and seven of the 22 genes encoding enzymes, indicating that the network may vary over the course of a growth season. We observed that all of the earlywood genes were positively connected to *PgNAC‐7* and the latewood genes were negatively connected to *PgNAC‐7*.

A network was constructed for September preferential genes. The most connected hub genes were three stress‐responsive genes, that is, a nucleotide binding site–leucine‐rich repeat (NBS‐LRR)‐encoding gene (activated disease resistance 1 (*ADR1*)), a dehydrin‐encoding gene (*DHR*), and a tubby protein‐encoding gene (*TUB*) (Tables 3b, S1). Several other dehydrins were also coregulated in this network (Table S1). These findings are consistent with changes in environmental factors, including water deficit and temperature decrease, that occur at the end (September) of a growth season and influence latewood formation in conifers.

## Discussion

Our findings describe the modular organization of the white spruce transcriptome, which we divided into invariant genes and coexpression groups of variable genes across tissues. We assigned 18 052 genes to 22 coexpression groups that are statistically robust and identified 4805 genes as being invariant across tissues. We also showed the complexity, conservation and reorganization of the transcriptome associated with secondary vascular tissue formation in spruces. These findings relating to transcriptome organization are discussed in the context of plant evolution, gene functions and expression networks.

### Evolutionary signatures of tissue differentiation

We measured transcript levels in seven vegetative tissues of above‐ and belowground organs of white spruce using a microarray platform and found that a large proportion of genes were differentially expressed across tissues with strong (10 548 high‐confidence genes) or moderate statistical support (7504 low‐confidence genes). Tissue differentiation has generally been linked to deeper transcriptome reorganization than developmental stages or environmental conditions in plants and animals. Our results from the comparison of white spruce tissues identified a much larger proportion of variable genes than comparisons of biological conditions or developmental stages in conifer trees, including interior hybrid spruce (Verne *et al*., 2011), maritime pine (Paiva *et al*., 2008), radiata pine (Li *et al*., 2010) and white spruce (this work). Similar findings have been reported in other plants including *A. thaliana* (Ma *et al*., 2005), tobacco (Edwards *et al*., 2010) and maize (Downs *et al*., 2013). In *A. thaliana*, Aceituno *et al*. (2008) indicated that tissue type was a more influential variable for transcriptome variation than any of the tested experimental conditions. In mammals, tissue affiliation explained generally at least 10 times more expression variance than perturbations or diseases (Prasad *et al*., 2013). In the mouse, Su *et al*. (2002) identified 78% of genes with differential expression across tissues vs 9–10% across time‐points (Kwon *et al*., 2012) or 1–7% between influenza‐infected lungs and control (Pommerenke *et al*., 2012). Tissue differentiation is evolutionarily ancient and has been linked to transcriptome signatures that are conserved across species (Prasad *et al*., 2013). In our previous work, we found that only 60 of the 5407 vascular tissue differential genes had different expression patterns when comparing three spruce species (Raherison *et al*., 2012). These findings demonstrate the way in which transcriptome signatures highlight the ancient evolutionary origins of tissue differentiation (*c*. 380 million yr ago; Kenrick & Crane, 1997).

In light of these observations and the high level of genomic conservation among spruces (Ritland *et al*., 2011), expression profiles may be regarded as a fundamental property of genes and their orthologues across the genus. It is also to be expected that profiles will extend to other genera within the Pinaceae by extrapolation from comparison of white spruce and loblolly pine showing a high level of conservation in gene coding sequences (Rigault *et al*., 2011) and in genome structure (Pavy *et al*., 2013). The response to abiotic stress factors is also well conserved between pine and spruce, as shown by Yeaman *et al*. (2014), who reported that the expression pattern was conserved for 74% of genes identified as sequence orthologues.

### Modular transcriptome organization underpins tissue differentiation

The features of sequences that make up the different tissue transcriptomes largely overlap but their quantitative variations in transcript amounts reveal the underlying transcriptome reorganization. These quantitative changes are underpinned by a complex modular organization that was represented here by coexpression groups (Figs 2, S2; Tables S3, S4). The modular organization may be illustrated by considering secondary xylem and phelloderm from the stem. Transcripts were detected for 22 970 and 23 296 genes in xylem and phelloderm, respectively, and were distributed among invariant genes and all of the 22 coexpression groups (Table S8). This modular organization showed the expressional diversity of gene profiles in a given tissue.

Tissue preferential expression has been used to classify genes, and to carry out functional studies and evolutionary investigations of tissue differentiation (Ma *et al*., 2005; Pavy *et al*., 2008; Yang & Wang, 2013; Duval *et al*., 2014). We analysed xylem and phelloderm preferential genes conserved in three spruce species and found that they were distributed among all coexpression groups as well as being differentially represented in many of them (Fig. 4). These observations show the diversity of expression profiles among genes identified as preferentially expressed in a given tissue.

### Coexpression is associated with functional similarity

We report a high rate of GO term enrichment among coexpression groups. Almost 50% of the genes with assigned GO terms were associated with GO term enrichment in the different groups (Figs 3a, S3). Coexpression groups were for the most part very distinct in regard to predominant enriched biological functions. Taken together, these observations indicated that several members within coexpression groups shared functions in addition to expression profiles, which supports the notion that members of coexpression groups participate in similar or common processes. For example, enriched terms for photosynthesis, stress response and lipid metabolic processes were largely observed in the group of genes highly expressed in foliage and shoot apex tissues (M1a in Fig. 3b; Table S2).The shoot apex requires lipids to support intense cell division activity and a high growth rate (Jordy, 2004). Both shoot apex and needles are photosynthetic tissues and are regularly exposed to stress conditions. In turn, cell division, cell/organ development and cell transduction were the predominant enriched functions in profiles with higher expression in root vascular tissues (M1b in Fig. 3b; Table S2). Root tips contain a meristematic zone in an active state of cell division (Reddy & Chary, 2003; Verbelen *et al*., 2006); root growth and development are controlled by cell signal transduction pathways (Perrin *et al*., 2005; Grieneisen *et al*., 2007; Laskowski *et al*., 2008).

Coexpression groups with opposite profiles within an expression module may have contrasting GO term enrichments (Figs 2a, S2). For example, the group with strong expression in secondary xylem tissues (M2a) had higher representation of terms related to cell/organ development than the group with low expression in secondary xylem (M2b). The opposite was observed for the terms related to carbohydrate/lipid reserves and defence mechanisms. Coexpression groups with high (M7a) and low (M7b) expression in root tips and xylem were enriched for secondary metabolic processes and for basic cellular mechanisms, respectively; and both were enriched for stress responsive genes.

Considering these observations, enriched GO terms may represent indirect information on the biological function of coexpressed genes which lack GO terms or functional annotations (i.e. guilt by association). Genes highly coexpressed have a greater chance of sharing a common regulatory mechanism and therefore have similar functions (Allocco *et al*., 2004). In this regard, a few of the coexpression profiles had a relatively low proportion of genes with associated GO terms (e.g. M3b and M11b) where < 25% of genes with assigned GO terms were associated with enriched GO terms. Genes without predicted functions are numerous in conifers (Rigault *et al*., 2011) and represent significant numbers even in model organisms. Coexpression groups may help not only to functionally annotate these genes but also to improve inference of gene function across species. According to Prasad *et al*. (2013), an analysis combining tissue expression profiles with sequence similarity provides a better prediction of functions of homologous genes across species than using sequence information alone.

We identified 4805 genes that did not vary across tissues (invariant genes) and thus could be designated as housekeeping genes according to Chang *et al*. (2011) and Eisenberg & Levanon (2013). The number of putative housekeeping genes decreases to 1201 when using expression level criteria (moderate to high expression genes; Table 2) defined by Aceituno *et al*. (2008) in *A. thaliana*. We found about the same number of housekeeping genes in white spruce as reported for humans (Chang *et al*., 2011) and three‐fold more than reported for *A. thaliana* (Aceituno *et al*., 2008), both of which studies considered many more tissue types and biological conditions. As relevant for housekeeping functions, the majority of invariant genes had a biological function related to basic cellular activities, in agreement with previous reports (Chang *et al*., 2011; Eisenberg & Levanon, 2013). Based on sequence similarity analysis, we also found that most of the highly expressed invariant genes were conserved between *A. thaliana* and white spruce (Table 2). This finding is consistent with previous reports indicating high expression of the majority of genes conserved between closely (mouse and human; Liao & Zhang, 2006) or distantly related species (mouse and fruit fly; Tamura *et al*., 2004). Highly expressed genes were shown to have lower evolutionary rates of change in protein sequences (Tamura *et al*., 2004; Liao & Zhang, 2006) and to be under stronger selection for mRNA folding (Park *et al*., 2013).

### Temporal reorganization of gene expression networks in the vascular transcriptome

Network analysis identified *PgNAC‐7* as a network hub that is positively connected to several genes involved in secondary cell wall formation. These findings are consistent with the role of *PgNAC‐7* and downstream MYB genes (*PgMYB1*, ‐*2*, ‐*4* and ‐*8*) which have been shown in white spruce to act as secondary cell wall regulators in transient assays (Duval *et al*., 2014) and in transgenic trees (Bomal *et al*., 2008). This network is functionally similar to that regulated by class IIB NAC proteins VND4, VND5 and VND6 of *A. thaliana* (Duval *et al*., 2014) and several downstream genes of MYBs (Zhong & Ye, 2009; Zhong *et al*., 2010), which is also conserved in woody angiosperms including poplar (*Populus trichocarpa*) and eucalyptus (*Eucalyptus gunnii*) (Zhong & Ye, 2009; Zhong *et al*., 2010; Hussey *et al*., 2013).

Temporal analysis in white spruce indicated that *PgNAC‐7* and three of the spruce MYBs had expression profiles strongly related to earlywood, while no temporal variation was observed for other MYBs and most of the downstream genes encoding enzymes involved in secondary cell wall assembly. These observations indicate that the regulatory network may be reconfigured over the course of a growth season. A scan of the latewood preferential cluster (Table S1) identified one other NAC sequence and four other MYBs but these sequences have not been tested functionally. In turn, they represent candidate genes to be investigated for involvement in the regulation of members of this network.

Remodelling of the regulatory network in latewood was supported by the finding that the most connected hubs are stress responsive genes, that is, *ADR1*, a tubby gene and a dehydrin gene. *ADR* genes encode NBS‐LRR proteins involved in receptor‐mediated signalling (Bonardi *et al*., 2011). The network also contained many other coregulated dehydrin genes. ADR1, tubby protein and dehydrin proteins enhance drought tolerance in plants (Chini *et al*., 2004; Puhakainen *et al*., 2004; Wardhan *et al*., 2012). Our findings are consistent with the transition to dormancy that is initiated during latewood formation and involves several cellular changes including shifts in osmotic processes (Paiva *et al*., 2008). Differences between earlywood and latewood formation in terms of biological function and transcriptional regulatory network indicate transcriptional changes associated with seasonal network reorganization during a growing season in conifers.

### Concluding remarks

We classified expressed white spruce genes into coexpression groups that are indicative of the modular organization of the transcriptome. Our results showed that deeper transcriptome reorganization is associated with tissue differentiation than with developmental stages or environmental conditions, and that patterns are conserved between spruce species, as might be expected given the ancient evolutionary origins of tissue differentiation. Time‐course analyses of wood formation indicated network reorganization and a transition from cell wall formation towards defence‐related genes. This report extends previous work on the PiceaGeneExpress database (Raherison *et al*., 2012) by statistical modelling of transcriptome organization leading to network analysis. We have developed a framework of information for spruce genes and an approach that could be readily applied to any number of tree and other plant genomes. Combined with other types of information, coexpression groups may enable basic discoveries to be made relating to genome function, regulatory networks, biomarkers and candidate genes in addition to supporting the development of applications for breeding and the conservation of genetic diversity.

## Supporting information

Please note: Wiley Blackwell are not responsible for the content or functionality of any supporting information supplied by the authors. Any queries (other than missing material) should be directed to the *New Phytologist* Central Office.

**Fig. S1** Hierarchical clustering of white spruce (*Picea glauca*) tissues based on variable genes.
**Fig. S2** Representation of coexpression groups and their correlation to white spruce (*Picea glauca*) tissues.
**Fig. S3** Functional annotation of invariant genes and coexpression groups.
**Fig. S4** Gene degree distribution on a double log scale of the xylem (M2‐7) preferential network.

**Table S1** Compilation of gene expression results from Expts 1 and 2, gene network analysis and annotation summary
**Table S2** Functional annotation analysis of gene groups
**Table S3** Reverse transcription quantitative PCR (RT‐qPCR) primer sequences and microarray validation by RT‐qPCR
**Table S4** Number of genes per coexpression group in white spruce (*Picea glauca*)
**Table S5** Under‐ or overrepresentation of conifer‐specific genes across coexpression groups
**Table S6** Enriched pathways in xylem (M2–7) preferential network
**Table S7** Members of the secondary cell wall gene network shown to be transactivated by PgNAC‐7 or MYB transcription factors in white spruce (*Picea glauca*)
**Table S8** Distribution of tissue‐detected genes across invariant and coexpression groups of white spruce (*Picea glauca*)
**Methods S1** Weighted correlation network analysis (wgcna) script.
