# Supplementary material for: Modular organization of the white spruce (Picea glauca) transcriptome reveals functional organization and evolutionary signatures
Source: New Phytol. 2015 Feb 27;207(1):172–87. doi: 10.1111/nph.13343 (PMC5024012; doi:10.1111/nph.13343)
Supplement: Supplementary file 1 — Fig. S1 Hierarchical clustering of white spruce (Picea glauca) tissues based on variable genes. Fig. S2 Representation of coexpression groups and their correlation to white spruce (Picea glauca) tissues. Fig. S3 Functional annotation of invariant genes and coexpression groups. Fig. S4 Gene degree distribution on a double log scale of the xylem (M2‐7) preferential network. [file NPH-207-172-s001.pdf]

Supporting Information Figs S1–S4, Tables S4, S7 & S8 and Methods S1

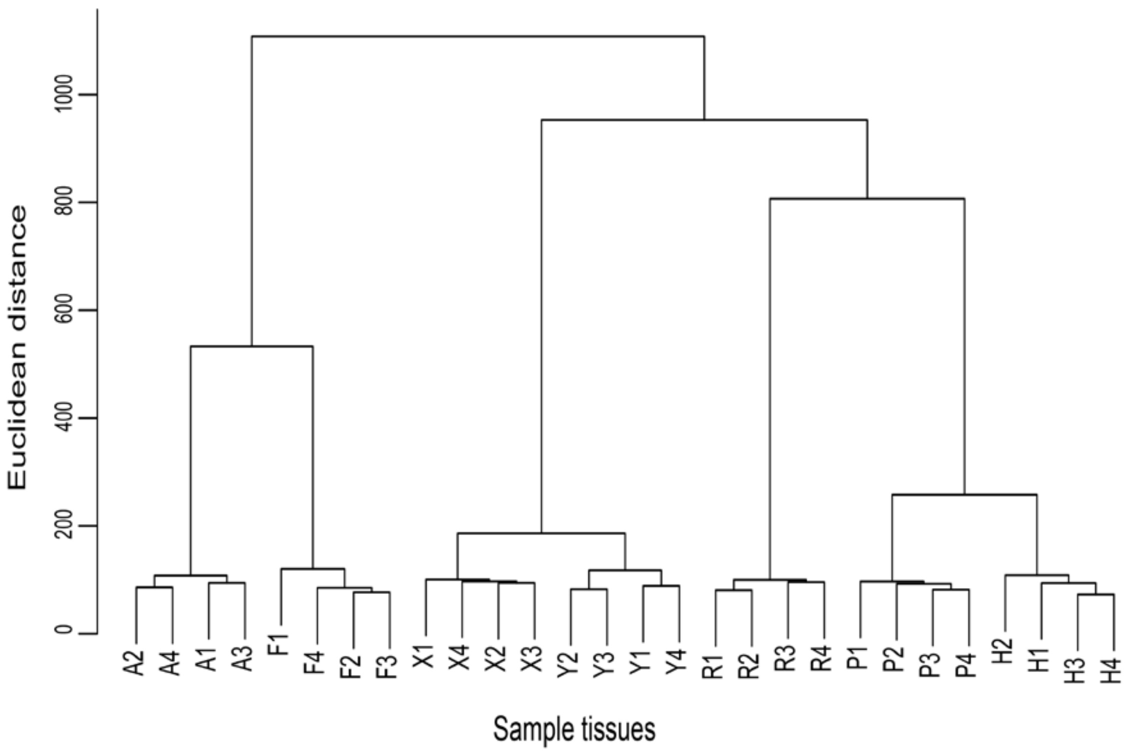

Fig. S1 Hierarchical clustering of white spruce (*Picea glauca*) tissues based on variable genes. Analysis was performed using the *hclust* function of R with Ward's method (Ward, 1963) and microarray data (log_2 scale) of variable genes. A, shoot apex; F, young foliage; X, shoot secondary xylem; Y, root secondary xylem; R, root tips; P, shoot phelloderm; H, root phelloderm. Each tissue was replicated four times (1–4).

Reference

Ward JH. 1963. Hierarchical grouping to optimize an objective function. *Journal of the American Statistical Association* **58:** 236–244.

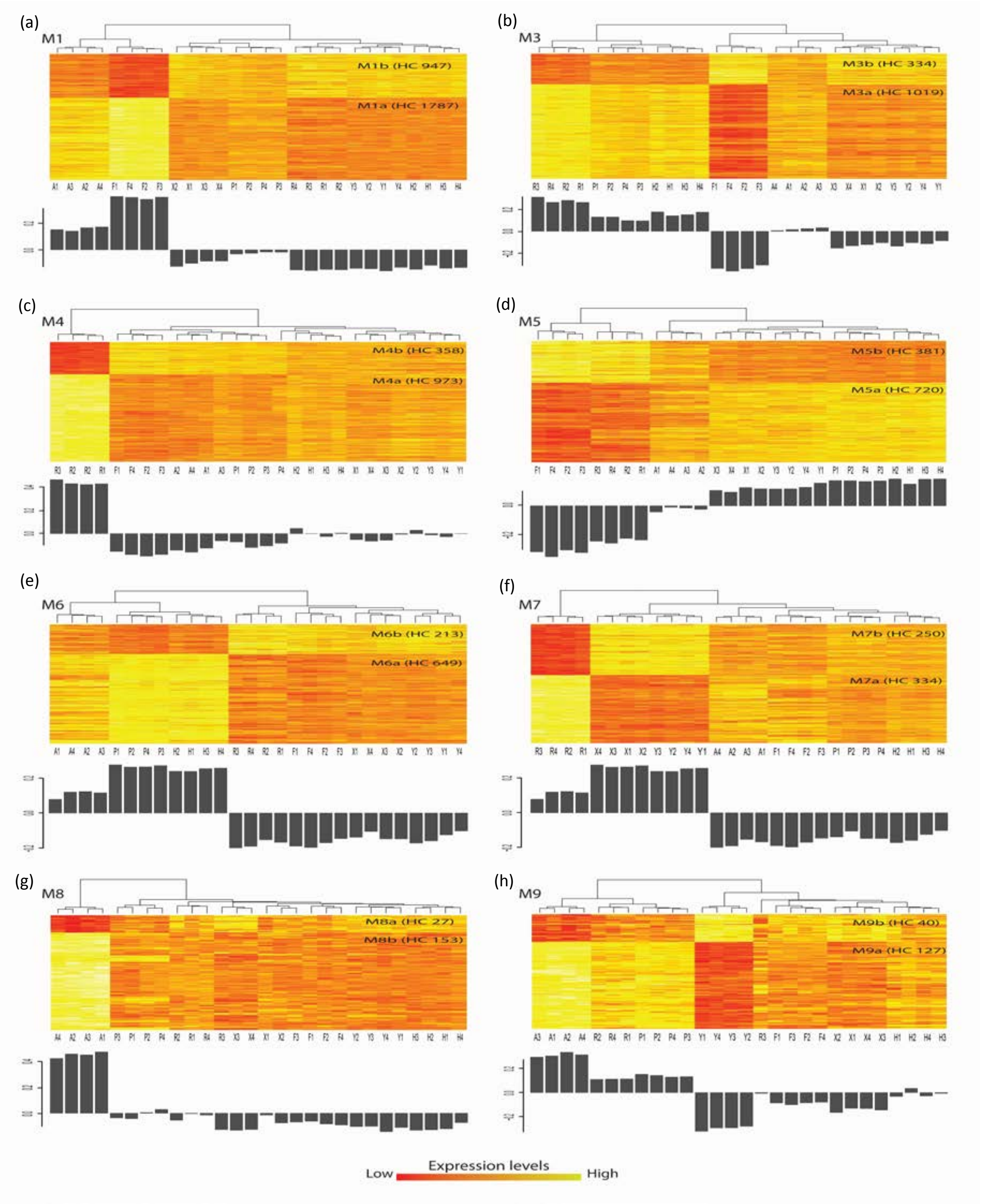

Fig. S2 Representation of coexpression groups and their correlation to white spruce (*Picea glauca*) tissues. M1 and M3–M9, expression modules; M1a,b and M3a–M9b, coexpression groups. (a–h) Heatmaps show transcript abundance (log_2 scale) of high-confidence (HC) variable genes in expression modules and coexpression groups determined with the *WGCNA* package of R. Number in parentheses, number of high-confidence variable genes in the coexpression group. Total and low-confidence variable

gene numbers are listed in Table S2. Rows (*y*-axis), genes which are listed in Table S1; columns (*x*-axis), tissues (A, shoot apex; P, shoot phelloderm; H, root phelloderm; R, root tips; F, young foliage; X, shoot secondary xylem; Y, root secondary xylem). Each tissue was represented by four biological replicates (1–4). Bar plots display the module eigengenes (or the first principal components).

(i)

(j)

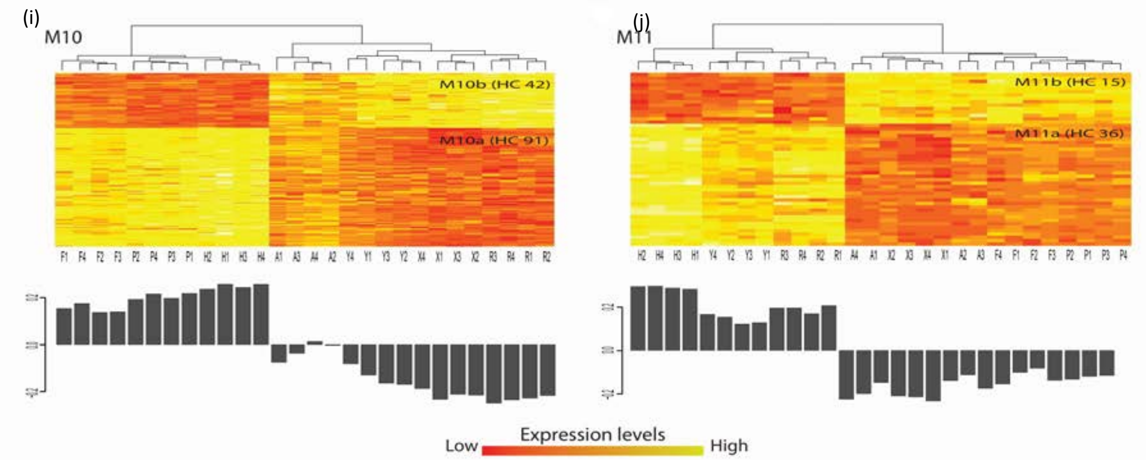

(k)

	M5b	M7a	M2b	M9a	M8b	M1a	M4b	M11b	M3b	M6a	M10a	M4a	M3a	M1b	M11a	M8a	M6b	M10b	M5a	M7b	M2a	M9b
R1	0,52	0,76	0,08	-0,01	-0,26	-0,36	-0,93	-0,61	-0,55	-0,38	-0,46	0,95	0,56	0,37	0,60	0,55	0,55	0,55	-0,47	-0,80	-0,08	-0,12
R4	0,54	0,78	0,11	0,02	-0,24	-0,34	-0,92	-0,59	-0,55	-0,37	-0,45	0,94	0,56	0,35	0,59	0,53	0,54	0,54	-0,49	-0,82	-0,11	-0,15
R2	0,52	0,82	0,17	0,11	-0,16	-0,33	-0,91	-0,56	-0,62	-0,29	-0,41	0,94	0,62	0,34	0,57	0,46	0,46	0,51	-0,47	-0,86	-0,17	-0,24
R3	0,48	0,74	0,05	-0,05	-0,28	-0,40	-0,94	-0,65	-0,55	-0,37	-0,47	0,96	0,57	0,41	0,63	0,58	0,55	0,56	-0,43	-0,77	-0,05	-0,08
A1	0,24	0,23	0,53	0,89	0,86	0,66	0,45	0,80	0,04	0,26	0,21	-0,40	-0,10	-0,64	-0,65	-0,91	-0,39	-0,20	-0,26	-0,23	-0,57	-0,84
A3	0,21	0,27	0,59	0,92	0,85	0,61	0,43	0,74	-0,07	0,36	0,28	-0,36	0,00	-0,59	-0,58	-0,89	-0,47	-0,26	-0,21	-0,27	-0,62	-0,88
A4	0,17	0,20	0,60	0,89	0,81	0,66	0,52	0,78	0,00	0,38	0,36	-0,46	-0,08	-0,65	-0,62	-0,90	-0,52	-0,35	-0,18	-0,19	-0,63	-0,83
A2	0,20	0,26	0,62	0,92	0,82	0,64	0,46	0,74	-0,06	0,38	0,34	-0,40	-0,02	-0,62	-0,57	-0,89	-0,50	-0,32	-0,21	-0,25	-0,66	-0,87
F1	0,72	0,17	0,46	0,25	0,20	0,95	0,48	0,50	0,62	-0,42	0,20	-0,45	-0,75	-0,96	-0,50	-0,29	0,24	-0,22	-0,75	-0,11	-0,46	-0,17
F4	0,72	0,19	0,50	0,28	0,20	0,96	0,49	0,51	0,59	-0,38	0,24	-0,45	-0,73	-0,96	-0,50	-0,30	0,20	-0,25	-0,75	-0,13	-0,51	-0,20
F3	0,70	0,24	0,58	0,42	0,32	0,98	0,51	0,57	0,51	-0,28	0,28	-0,46	-0,65	-0,98	-0,53	-0,41	0,11	-0,28	-0,73	-0,19	-0,59	-0,34
F2	0,68	0,17	0,52	0,32	0,26	0,98	0,54	0,55	0,58	-0,32	0,27	-0,50	-0,72	-0,98	-0,53	-0,37	0,14	-0,29	-0,71	-0,11	-0,53	-0,24
H4	-0,51	0,00	0,25	-0,08	-0,32	-0,47	-0,06	-0,55	-0,64	0,79	0,62	0,13	0,58	0,47	0,67	0,27	-0,66	-0,58	0,59	0,00	-0,21	0,06
H1	-0,46	0,04	0,28	-0,09	-0,36	-0,44	-0,06	-0,57	-0,63	0,76	0,64	0,14	0,56	0,44	0,69	0,31	-0,64	-0,60	0,54	-0,03	-0,25	0,07
H2	-0,50	0,01	0,15	-0,20	-0,43	-0,57	-0,18	-0,67	-0,64	0,68	0,52	0,23	0,60	0,57	0,75	0,41	-0,54	-0,48	0,58	-0,01	-0,11	0,17
H3	-0,53	-0,03	0,23	-0,10	-0,33	-0,46	-0,03	-0,54	-0,62	0,79	0,63	0,10	0,55	0,46	0,65	0,27	-0,67	-0,60	0,61	0,04	-0,20	0,08
P1	-0,43	-0,01	0,54	0,52	0,24	0,05	0,42	0,20	-0,46	0,95	0,79	-0,33	0,35	-0,05	-0,02	-0,42	-0,97	-0,78	0,47	0,04	-0,54	-0,46
P2	-0,43	-0,01	0,54	0,56	0,30	0,08	0,44	0,25	-0,44	0,95	0,76	-0,35	0,34	-0,07	-0,06	-0,48	-0,97	-0,76	0,46	0,03	-0,54	-0,50
P4	-0,41	-0,06	0,53	0,57	0,36	0,16	0,53	0,34	-0,35	0,92	0,77	-0,44	0,25	-0,16	-0,16	-0,56	-0,97	-0,77	0,44	0,09	-0,53	-0,51
P3	-0,44	-0,10	0,50	0,54	0,32	0,14	0,54	0,33	-0,33	0,92	0,77	-0,45	0,24	-0,14	-0,16	-0,53	-0,97	-0,78	0,47	0,13	-0,51	-0,47
Y4	-0,43	-0,61	-0,86	-0,94	-0,60	-0,58	-0,20	-0,46	0,33	-0,38	-0,43	0,09	-0,19	0,56	0,27	0,62	0,44	0,37	0,40	0,59	0,89	0,92
Y1	-0,41	-0,61	-0,92	-0,90	-0,52	-0,58	-0,22	-0,38	0,35	-0,44	-0,54	0,10	-0,19	0,57	0,19	0,55	0,49	0,48	0,38	0,58	0,94	0,87
Y2	-0,33	-0,54	-0,91	-0,89	-0,52	-0,57	-0,29	-0,41	0,35	-0,51	-0,60	0,17	-0,19	0,55	0,21	0,58	0,57	0,54	0,29	0,51	0,93	0,86
Y3	-0,36	-0,60	-0,92	-0,90	-0,51	-0,53	-0,21	-0,36	0,40	-0,50	-0,57	0,09	-0,24	0,52	0,15	0,54	0,54	0,51	0,32	0,57	0,93	0,88
X2	-0,36	-0,68	-0,96	-0,73	-0,29	-0,40	-0,08	-0,03	0,54	-0,54	-0,66	-0,06	-0,36	0,38	-0,18	0,29	0,53	0,58	0,29	0,65	0,96	0,74
X3	-0,32	-0,71	-0,93	-0,67	-0,21	-0,27	0,04	0,10	0,64	-0,55	-0,63	-0,17	-0,46	0,26	-0,31	0,18	0,52	0,55	0,25	0,69	0,92	0,70
X4	-0,39	-0,77	-0,92	-0,66	-0,20	-0,25	0,11	0,15	0,65	-0,48	-0,57	-0,25	-0,48	0,24	-0,35	0,13	0,43	0,47	0,31	0,75	0,91	0,69
X1	-0,37	-0,72	-0,93	-0,58	-0,10	-0,27	0,06	0,19	0,60	-0,49	-0,65	-0,19	-0,42	0,26	-0,38	0,07	0,45	0,56	0,29	0,69	0,92	0,60

-1 Pearson correlation coefficient +1

Fig. S2 (continued) Representation of coexpression groups and their correlation to white spruce (*Picea glauca*) tissues. M10 and M11, expression modules; M10a–M11b, coexpression groups. (i, j) Heatmaps show transcript abundance ($\log_2$ scale) of high-confidence (HC) variable genes in expression modules and coexpression groups determined with the *WGCNA* package of R. Number in parentheses, number of high-confidence variable genes in the coexpression group. Total and low-confidence variable gene numbers are listed in Table S2. rows (*y*-axis), genes which are listed in Table S1;

columns (*x*-axis), tissues (F, young foliage; P, shoot phelloderm; H, root phelloderm; A, shoot apex; Y, root secondary xylem; X, shoot secondary xylem; R, root tips). Each tissue was represented by four biological replicates (1–4). Bar plots display the module eigengenes (or the first principal components). (k) Heatmap shows the Pearson correlation coefficient between coexpression groups and tissue samples.

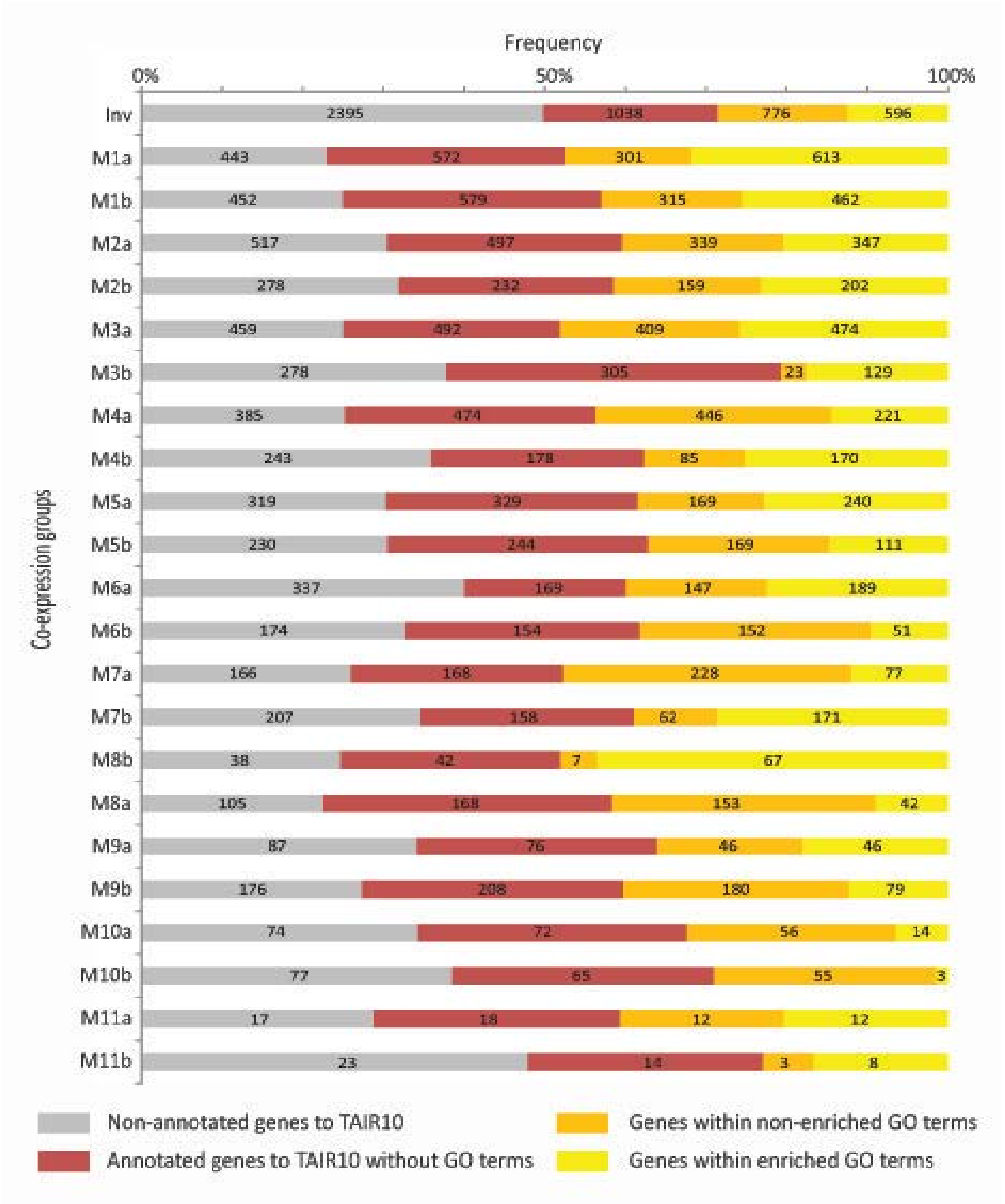

Fig. S3 Functional annotation of invariant genes and coexpression groups. Inv, invariant genes; M1a–M11b, coexpression groups. *x*-axis, frequency (%) of genes that were associated to each of the four annotation categories: non-annotated genes to The *Arabidopsis* Information Resource version 10 (TAIR10, www.arabidopsis.org); annotated genes to TAIR10 but without gene biological process gene ontology (GO) terms; genes within enriched and non-enriched GO terms. Numbers inside each bar are the numbers of

genes in each category. Enrichment test was performed using Database for Annotation, Visualization and Integrated Discovery (DAVID) tool (http://david.abcc.ncifcrf.gov, Huang *et al*., 2009).

Reference

Huang DW, Sherman BT, Lempicki RA. 2009. Bioinformatics enrichment tools: paths toward the comprehensive functional analysis of large gene lists. *Nucleic Acids Research* **37:** 1–13.

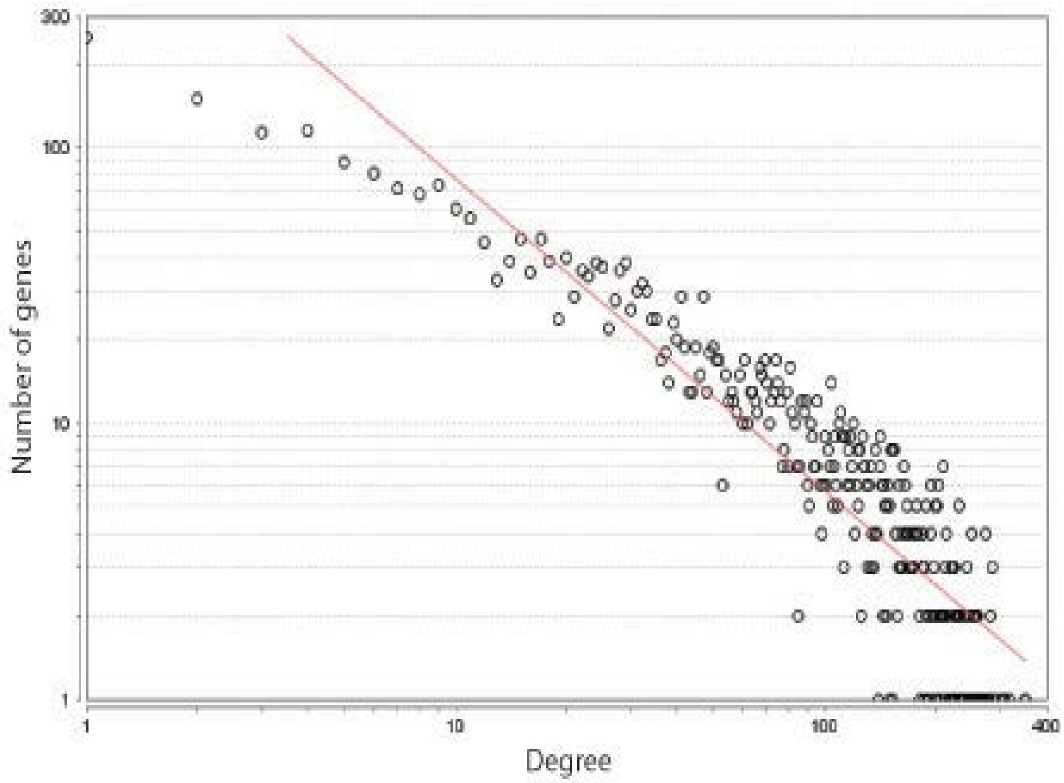

Fig. S4 Gene degree distribution in a double log scale of the xylem (M2–7) preferential network. The M2–7 network had a scale-free topology since its gene degree distribution fitted to a power-law distribution (i.e. few hubs hold together numerous small-degree nodes; Barabási *et al*., 2001). Figure as output directly from the Cytoscape software (Assenov *et al.*, 2008). June–July and September preferential networks were not scale free.

References

Assenov Y, Ramírez F, Schelhorn S, Lengauer T, Albrecht M. 2008. Computing topological parameters of biological networks. *Bioinformatics* **24:** 282–284.

Barabasi AL, Erzsebet R,Vicsek T. 2001. Deterministic scale-free networks. *Physica A* **299:** 559–564.

Table S4 Number of genes per coexpression group in white spruce (*Picea glauca*)

**Coexpression group**	**High-confidence gene**	**Low-confidence gene**	**Total**
M1a	1787	142	1929
M1b	947	861	1808
M2a	1418	282	1700
M2b	628	243	871
M3a	1019	815	1834
M3b	334	401	735
M4a	973	553	1526
M4b	358	318	676
M5a	720	333	1053
M5b	381	373	754
M6a	649	193	842
M6b	213	318	531
M7a	334	305	639
M7b	250	348	598
M8a	153	1	154
M8b	27	441	468
M9a	127	128	255
M9b	40	603	643
M10a	91	125	216
M10b	42	158	200
M11a	36	23	59
M11b	15	33	48

High- and low-confidence genes are genes differentially expressed across tissues at adj$P$-value < 0.0001 and 0.05 < adj$P$-value > 0.0001, respectively. M1a–M11b are tissue-based coexpression groups that are graphically presented in Fig. 2(a) and Fig. S2(a–j).

Table S7 Members of the secondary cell wall gene network shown to be transactivated by PgNAC-7 or MYB transcription factors in white spruce (*Picea glauca*)

GenBank Accession No.	Cluster ID[1]	Gene name[2]	Function	Trans activation[3]	PgNAC-7 (R>0.9)[4]	PgMyb8 (R>0.9)[4]	Coexpression group	Temporal cluster[5]	Preferential expression in spruces[6]
BT102049	GQ0165_B14	PgNAC-7	NAC-domain transcription factor			x	M2a	T2a	Xylem
BT108136	GQ03117_E18	PgMYB8	myb domain protein	*a*, *b*	x		M2a	None	Xylem
BT108631	GQ03124_J09	PgMYB1	myb domain protein	*a*, *b*			M3b	T2a	Xylem
BT108414	GQ03121_H22	PgMYB2	myb domain protein	*b*	x		M2a	T2a	Xylem
BT107883	GQ03113_N22	PgMYB4	myb domain protein	*b*	x		M2a	T2a	Xylem
BT116706	GQ03805_C07	PgDHS-2	3-deoxy-d-arabino-heptulosonate 7-phosphate synthase	*a*, *b*	x		M2a	None	Xylem
BT115945	GQ03709_D11	PgCM-3	Chorismate mutase-1	*b*	x		M2a	None	Xylem
BT112280	GQ03312_O11	PgCAD	cinnamyl alcohol dehydrogenase	*a*, *b*	x	x	M2a	None	Xylem
BT106671	GQ03007_K12	Pg4CL	4-coumarate:CoA ligase	*a*, *b*			M2a	None	Xylem
BT111802	GQ03303_N02	PgCCR-1	cinnamoyl coa reductase	*b*	x		M2a	None	Xylem
BT111350	GQ03236_G10	PgPRR-2	pinoresinol reductase	*b*	x	x	M2a	None	Xylem
BT116976	GQ03810_K09	PgCesA-3	Cellulose synthase family protein	*a*, *b*	x		M2a	T1b	Xylem
BT106155	GQ02904_A20	PgTUA-1	tubulin alpha-4 chain	*a*, *b*			M6a	None	None
BT116258	GQ03714_M12	PgXTH8-1	xyloglucan endotransglucosylase / hydrolase	*a*, *b*			M4a	T1b	Phelloderm

[1]Rigault *et al.* (2011); [2]Duval *et al.* (2014). [3]Functional evidence of transcriptional control was obtained from *a*, transient cotransfection with PgNAC-7, PgMYB-8 or both (Duval *et al.*, 2014) or *b*, stable overexpression of PgMYB8 (Bomal *et al.*, 2008). [4]Correlations with PgNAC-7 and PgMYB-8 from network analyses. [5]Temporal cluster T2a and T1b were highly and moderately June–July preferential genes, respectively; none indicates no temporal variation detected. [6]Raherison *et al.* (2012); conserved preferential expression in three spruces (*P. glauca*, *P. abies*, *P. sitchensis*) comparing stem secondary xylem and phelloderm tissues determined by microarray.

References

Bomal C, Bedon F, Caron S, Mansfield SD, Levasseur C, Cooke JE, Blais S, Tremblay L, Morency M-J, Pavy N. 2008. Involvement of *Pinus taeda* MYB1 and MYB8 in phenylpropanoid metabolism and secondary cell wall biogenesis: a comparative in planta analysis. *Journal of Experimental Botany* **59:** 3925–3939.

Duval I, Lachance D, Giguère I, Bomal C, Morency M-J, Pelletier G, Boyle B, MacKay JJ, Séguin A. 2014. Large-scale screening of transcription factor–promoter interactions in spruce reveals a transcriptional network involved in vascular development. *Journal of Experimental Botany* **65:** 2319–2333.

Raherison E, Rigault P, Caron S, Poulin P-L, Boyle B, Verta J-P, Giguère I, Bomal C, Bohlmann J, MacKay J. 2012. Transcriptome profiling in conifers and the PiceaGenExpress database show patterns of diversification within gene families and interspecific conservation in vascular gene expression. *BMC Genomics* **13:** 434.

Table S8 Distribution of detected genes across invariant gene and coexpression groups of white spruce (*Picea glauca*)

	**Foliage**	**Shoot apex**	**Shoot phelloderm**	**Shoot xylem**	**Root phelloderm**	**Root xylem**	**Root tips**
Inv	4554	4414	4638	4511	4606	4375	4492
M1a	1928	1925	1908	1882	1892	1818	1840
M1b	1797	1791	1807	1808	1807	1806	1806
M2a	1675	1671	1686	1700	1685	1696	1661
M2b	871	869	868	849	868	838	860
M3a	1807	1824	1834	1816	1833	1810	1831
M3b	734	733	730	732	726	728	715
M4a	1469	1472	1492	1472	1512	1471	1524
M4b	675	675	672	669	672	661	654
M5a	1042	1049	1052	1051	1053	1048	1032
M5b	754	748	743	733	738	725	749
M6a	831	839	842	830	842	819	822
M6b	524	519	526	521	522	523	530
M7a	623	632	631	609	633	594	636
M7b	591	591	596	598	597	597	584
M8a	139	154	145	136	134	105	124
M8b	467	465	467	467	467	467	468
M9a	249	255	254	247	254	237	252
M9b	637	633	641	641	640	642	634
M10a	215	212	216	210	216	210	210
M10b	196	196	196	198	196	191	199
M11a	57	54	58	53	59	57	59
M11b	48	48	48	48	47	46	46
Unclassified	500	502	506	496	497	487	492
Tested_genes	22383	22271	22556	22277	22496	21951	22220
Detected_genes	23104	22951	23296	22970	23225	22608	22913

Inv, invariant genes; M1a–M11b, coexpression groups; unclassified, variable genes that could not be classified to any coexpression groups using WGCNA or template matching methods; tested_genes, detected gene that met assumptions for ANOVA testing (for details, see the Materials and Methods section); detected_genes, genes whose transcript levels were detected above background levels in the tissue.

Methods S1 Weighted correlation network analysis (WGCNA) script.

WGCNA is an R package that can be briefly described as follow. A pair-wise Pearson correlation matrix was computed and then transformed into an adjacency matrix using a power function: adjacency value of two genes = $|\text{correlation between two genes}|^{\beta}$, where β is the weight. The adjacency matrix was transformed into a distance matrix using the topological overlap measure (Li & Horvath, 2007). A hierarchical clustering analysis was applied to the dissimilarity matrix (1 - distance matrix). Dendrogram leaves were cut and separated genes into distinct expression modules using the dynamic tree cut algorithm (Langfelder *et al*., 2008). An expression module can be defined as a cluster of highly (positively and negatively) correlated genes (Langfelder & Horvath, 2008). It reveals two negatively correlated expression profiles. Each expression profile is associated with a group of genes that we referred to a coexpression group in experiment 1 or temporally variable cluster in experiment 2 (Table S1). Below is the WGCNA script we used:

```
# Loading expression data
# Expression data is a matrix with genes in rows and tissues in columns
getwd();
workingDirectory="C:/Users/...";
setwd(workingDirectory);
library(WGCNA);
options(stringsAsFactors = FALSE);
datExp=read.table("ExpressionData.txt")

# Transposing data and removing unnecessary data
datExpr0 = as.data.frame(t(datExp[,]));
names(datExpr0)=datExpr0[1,]
datExpr0=datExpr0[-1,]

# Detecting outlier samples with hierarchical clustering analysis
# no sample of experiments 1 and 3 was removed after the outlier verification
sampleTree = flashClust(dist(datExpr0), method = "average");
plot(sampleTree)

# Choosing the soft-thresholding power: analysis of network topology
powers = c(c(1:10), seq(from = 12, to=20, by=2));
sft = pickSoftThreshold(datExpr0, powerVector = powers, verbose = 5);
plot(sft$fitIndices[,1], -sign(sft$fitIndices[,3])*sft$fitIndices[,2],
xlab="Soft Threshold (power)", ylab="Scale Free Topology Model Fit,
signed R^2",type="n", main = paste("Scale independence"));
text(sft$fitIndices[,1], -sign(sft$fitIndices[,3])*sft$fitIndices[,2],
labels=powers,cex=cex1,col="red");
```

```
abline(h=0.86,col="red")
plot(sft$fitIndices[,1], sft$fitIndices[,5],
xlab="Soft Threshold (power)",ylab="Mean Connectivity", type="n",
main = paste("Mean connectivity"))
text(sft$fitIndices[,1], sft$fitIndices[,5], labels=powers, cex=cex1,col="red")


# Identifying expression modules
#  We used power = 14 and 18 to identify modules across tissues (Experiment 1)
# and times (Experiment 3), respectively
net = blockwiseModules(datExpr0, power = 14, minModuleSize = 30,
maxBlockSize=12000, reassignThreshold = 0, mergeCutHeight = 0.25,
numericLabels = TRUE, pamRespectsDendro = FALSE, saveTOMs = TRUE,
saveTOMFileBase = "TOM1", verbose = 3)

# Calculating eigengene of modules
MEs = moduleEigengenes(datExpr0, moduleColors)$eigengenes
MET =orderMEs(MEs)
```

References

Langfelder P, Horvath S. 2008. WGCNA: an R package for weighted correlation network analysis. *BMC Bioinformatics* **9:** 559.

Langfelder P, Zhang B, Horvath S. 2008. Defining clusters from a hierarchical cluster tree: the Dynamic Tree Cut package for R. *Bioinformatics* **24:** 719–720.

Li A, Horvath S. 2007. Network neighborhood analysis with the multi-node topological overlap measure. *Bioinformatics* **23:** 222–231.
